# Pancreatic Neuroendocrine Diagnostic Imaging Order and Reader Evaluation over Two Decades in a Tertiary Academic Center

**DOI:** 10.3390/diagnostics15080960

**Published:** 2025-04-10

**Authors:** Sara Babapour, Annabel Chen, Grace Li, Luke Phan

**Affiliations:** Radiological Sciences, Clinical Research, David Geffen School of Medicine, University of California, Los Angeles, 10833 Le Conte Ave, Los Angeles, CA 90095, USA; annabelchen@mednet.ucla.edu (A.C.); graceli@mednet.ucla.edu (G.L.); lukephan@mednet.ucla.edu (L.P.)

**Keywords:** gastrointestinal radiology, pancreatic neuroendocrine neoplasms (panNENs), multi-modality imaging, pancreatic neuroendocrine tumor

## Abstract

**Background/Objective:** Identifying patterns of diagnostic imaging workflow parallel to the influence of certain variables, such as pathology guidelines over time, provides valuable insight for clinical decision making. This study presents a recurring trend of initial imaging orders and follow-ups, up to the diagnosis of pancreatic neuroendocrine tumors (pNETs), across two decades, with scans which led to pathological investigation. **Methods:** Three readers evaluated common conventional imaging among initial and follow-up studies for lesion detection and localization. Inter-reader and intra-reader analyses were controlled as contributing factors to the imaging diagnostic trend. **Results:** Our results show that CT was the prominent initial scan in pNET workup, likely due to their wide availability, high spatial resolution, and rapid acquisition, with a sufficient detection rate throughout both decades, regardless of technical advances. However, MRI scans also gained soaring popularity, especially among syndromic patients, likely due to follow-up and anatomical surgery precision. **Conclusions:** Newer modalities may be eventually useful and only requested for pNETs staging and further treatment.

## 1. Introduction

Pancreatic neuroendocrine tumors (pNETs) represent an indistinctly rare subset of neoplasms that develop from the endocrine cells of the pancreas. In 2021, 60,430 new pancreatic cancer cases were estimated, and 48,220 deaths were reported in the United States [1], with pNETs constituting 16% of all neuroendocrine tumors (NETs) [2]. NETs are distinguished by their ability to produce hormones from the more common pancreatic adenocarcinomas stemming from exocrine cells. However, distinguished pathological behavior in these tumors from carcinomas was discovered in 2000 and classified thereafter by WHO guidelines.

The pancreaticoduodenal region gives rise to specific functioning and well-characterized NET syndromes; however, their presentation may vary in clinical syndromes, combined with biochemical evidence of inappropriately elevated hormonal levels, defined as functional or syndromic NETs [3]. Meanwhile, some NETs may secrete biologically inactive hormonal variants or bioactive hormones at insufficient levels to elicit symptoms, and these should consequently be classified as nonfunctioning or non-syndromic NETs. The majority of pNET cases (50–85%) are nonfunctioning and present with more advanced diseases and a worse prognosis [4].

In rare cases, metachronous functioning syndromes marked by the simultaneous secretion of multiple bioactive hormones may occur in up to 3–6% of patients with pNETs during the progression of the disease, attributed to the extended complexity of these tumors [5].

Aligned with revolutionary advances in imaging techniques to distinguish these clinically challenging tumors, pathology studies after 2010 [6,7] integrated proliferation rates from mitotic counts in pNET classification to predict overall survival [8]. The early detection of pNETs is determined by a clinically inconsistent behavior, microscopic tumor size, location in the small pancreatic organ, low-contrast imaging, imaging heterogeneity from solid-to-cystic lesions, and overlapping clinical and imaging features. pNETs, ranging from slow-growing, indolent profiles to aggressive malignancies with varying degrees of disease activity, including growth rate, grade, differentiation, and metastatic potential [9], pose a great diagnostic challenge. Owing to their diverse nature in metabolically active hormonal origin or nonfunctional syndromes, these tumors necessitate a multidisciplinary approach, integrating the expertise of gastroenterologists, endocrinologists, oncologists, pathologists, and radiologists.

While pNET management is evolving according to refined pathology guidelines, surgical treatment algorithms, research developments in the radiology field, and an inclination towards using technologically advanced modalities could play a key role in replacing multiple cross-sectional modalities, such as computed tomography (CT), magnetic resonance imaging (MRI), and endoscopic ultrasound (EUS), with functional imaging techniques targeting somatostatin receptors (SSTRs), such as Octreotide single-photon emission computed tomography (SPECT) or SSTR PET/CT and PET/MRI using DOTA-peptides. Due to the relatively rare nature of pNETs, observing repetitive patterns in imaging workflow over two decades, along with subsequent patient outcomes provides valuable retrospective insights.

## 2. Methods and Materials

### 2.1. Target Population

This retrospective study focused on pNET patient population at UCLA (Los Angeles, USA), based on pathology data in a single tertiary-care academic center spanning two decades (1997–2021). All patients with either functional (insulinoma, gastrinoma, glucagonoma, VIPoma, carcinoid, unspecified) or nonfunctional tumors were identified using our tertiary institution’s surgical and pathological databases, and their pre-treatment imaging order requests were reviewed. The database included all patients with pathologically proven pNETs who later underwent fine-needle aspiration (FNA), core biopsy, or surgery, including their initial and follow-up imaging data. Demographic, clinical, histological, and surgical data were obtained. First, scan request trends were observed and categorized. Then, tumors were characterized by our radiology expert blinded readers, using conventional scans (CT and MRI).

**Figure 1:**
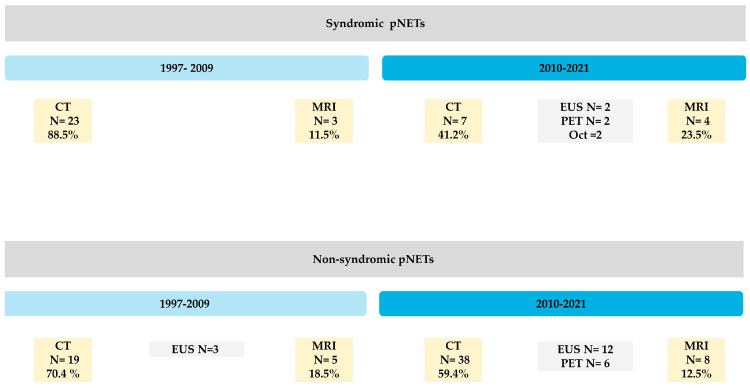
This figure shows overall multi-modality workflow over times for pancreatic neuroendocrine tumors detection at UCLA tertiary center with preference changes by tumor function.

Our two-decade pre-biopsy image database was aimed to define the radiology workflow trend (Figure 1) and to assess conventional imaging performance according to inter-reader and intra-reader studies.

### 2.2. Study Design

In this HIPAA-compliant study, we evaluated the imaging workup for patients with pNETs prior to surgical biopsy/FNA diagnosis. These patients were categorized as syndromic vs. non-syndromic, stratified by period to identify imaging order patterns. Primarily, 43 syndromic and 91 non-syndromic patients were identified over the two decades analyzed (Figure 1). Pre-biopsy CT, MRI, EUS, Oct, and PET scans and notes were recorded for each patient. Trends in radiologic workflow were assessed for patients with functional and nonfunctional pNETs between 1997 and 2021 before surgical biopsy or FNA diagnosis. The study cohort was stratified into syndromic and non-syndromic pNETs and compared across the two decades. Trends in imaging workflow were studied to extract the two most frequent conventional imaging techniques for further detection sensitivity evaluation by our readers. Patients with an identifiable specific pre-diagnosis clinical history had mostly undergone conventional imaging at some point in our institution and were selected for further assessment (among *N* = 75 patients). Following trend observation, three fellowship-trained abdominal radiologists, blinded to pathology report, reviewed the tumors on multidetector computed tomography (MDCT) and dynamic contrast enhancement (DCE) MRI, identified as the most common initial imagining modalities, localized and characterized the pNETs’ imaging features (e.g., calcification). Then detections were between readers (inter reader) and for random scans for each reader at different times (intra-reader) were evaluated for detection sensitivity and the impact of reader interpretation on the scan request trends on the most frequently used imaging modalities. Several subgroups outcomes were also extracted retrospectively based on the 2020 National Comprehensive Cancer Network (NCCN) guideline, which recommended surveillance over a period of 10 or more years, and the most recent American Joint Committee on Cancer (AJCC)’s updates on pancreatic tumor size (<2 cm, 2–4 cm, >4 cm). All data analyses were performed in compliance with the 1996 HIPAA Act, following the waiver of informed consent by the Institutional Review Board (IRB).

A descriptive overview of our pNET database spanning over two database is explained below: *

- ** Twenty-eight patients had medical history notes available but were only treated in our institution as a referral and follow-up. These patients were analyzed for establishing the trend according to the available multicenter notes but were not considered for further evaluation. Seventeen patients were primarily evaluated based on EUS data, eight patients were diagnosed with PET, and two with Oct, while one patient’s diagnosis was missed due to a stable lesion over a period of 4 years. Three patients had undergone their initial CT or MRI outside of our institution, without prior confirmatory documentation.

**Figure 2:**
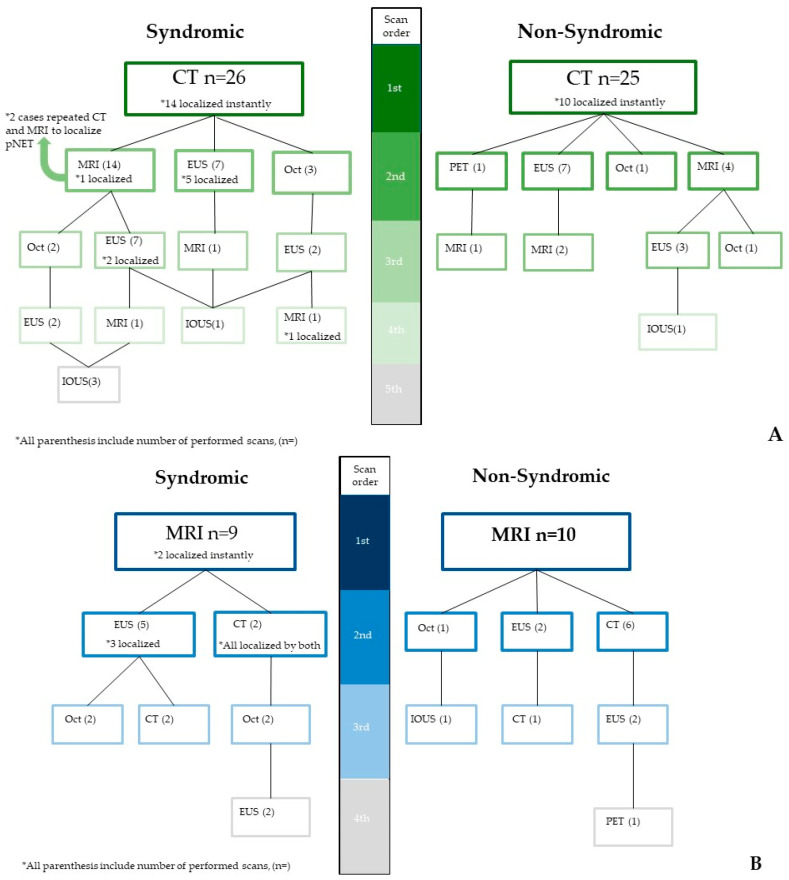
The two most commonly utilized modalities—CT (**A**) and MRI (**B**)—and their detection advantages in pNETs, with frequent pathways corresponding to clinical symptoms of patients. IOUS is an abbreviation for intra-operative ultrasound.

** Two patients had repeated both CT and MRI (*n* = 4 scans) before final tumor detection; therefore, latest scans prior to their surgery date were considered shown in Figure 2 (105 scans were considered instead of 107). In our cohort, *n* = 75 patients accounted for 105 CT or MR scans as their initial scans or follow-ups, while 4 CT and 7 MRI were inaccessible on our imaging archive platform to be reviewed by our experts (11/105). Three syndromic patients were diagnosed based on clinical findings prior to imaging, were still evaluated by our experts. To this end, the two most utilized conventional scans, CTs and MRIs, were transferred to our expert for a prospective reads.

## 3. Results

### 3.1. Our Data Characteristics

Our cohort study included CTs and MRIs of *N* = 75 patients, 55% of whom were female, with a mean age of 54.7 ± 17.23. The patients with pNETs were divided into 35 syndromic (24 detected up until 2010 and 11 detected after 2010) and 40 non-syndromic/nonfunctional pNET cases (21 detected before 2010 and 19 detected after 2010) (Table 1). The mean lesion size was 3.2 cm ± 2.7 (range: 0.3–11 cm). Two senior abdominal radiology fellows and one board-certified abdominal radiologist who were blinded to consensus reviewed morphologic imaging of our cases. A total of 58 CTs and 36 MRIs were reviewed per reader for characterization. For the syndromic patients, 27/36 initial scans (75%) were MDCT, and 9/36 cases (25%) used MRI. In non-syndromic patients, the final scan was CT in 24 follow-ups, MRI in 14 follow-up end-point images, and EUS in 11 follow-ups before treatment. Syndromic patients with a primary CT evaluation were subsequently undergone 36 more scans. MRI resulted in 11 follow-ups.

### 3.2. Inter-Reader and Intra-Reader Detection Assessment

Our reader 1 and 2 were clinical fellowship trained reviewers with 2+ years of experience; and reader 3 = fellowship attending with more than 10 years of experience.

The primary pNET tumor, with a mean lesion size of 3.2 cm +/− 2.7 (range: 0.3–11 cm) (Table 1), was successfully detected in 85% of cases by reader 1 (55 lesions in 58 CT scans and 28 lesions in 36 MRIs, with 12% false-negatives), 85% of times by reader 2, and 89% of cases by the attending. The detection sensitivity for pNETs was relatively high, with MDCT at 93% and DCE-MRI at 88% (CT had a 0.79 ICC, *p* < 0.0001, and MRI’s ICC was 0.58, *p* < 0.0001). Based on the symptom presentation category, the detection sensitivity rate for syndromic patients was 89% for MDCT and 80% for DCE MRI. For non-syndromic patients, the overall performance was 97% for CT and 100% for MRI.

Reader 1 accurately detected 83 cases overall (88% accuracy and 12% false-negative rate), including 55 out of 58 CTs (94%) and 28 out of 36 MRIs. Reader 2 accurately detected 80 cases overall (85% accuracy, 15% false-negative rate), including 51 positive findings in 58 CT scans and 29 true-positives in 36 MRIs. Following these results, our attending had an overall accuracy of 89% and an 11% false-positive rate. Our third expert had 93% accuracy in detection on CT (compared to 95% for reader 1 and 88% for reader 2). As for the MRI accuracy, it was 78%, 81%, and 83% for readers 1, 2, and 3, respectively. False-negative percentage was 5%, 12%, and 7% on CT, and for MRI, they were 22%, 19%, and 17% on MRI for reader 1, reader 2, and the attending radiologist, respectively. Reader agreeability among our fellows was 0.59 (Cohen’s kappa).

Our inter-reader analysis revealed excellent detection rates, with 31 concordant reads out of 36 MRI scans (86% agreement and sensitivity). Furthermore, 54 out of the 58 CT reads were compliant and concordant, with 93% sensitivity.

In our intra-reader assessments using repeated random scans, reader 1 suspected one lesion on a follow-up MRI scan (88% reproducibility of the result), and reader 2 had a repeated false-negative result on the same MRI scan, which could have been due to the lesion size and detection difficulty. Otherwise, no discrepancies were observed between the two reads among our radiologists.

**Theorem** **1.** *Regardless of advances in modalities, CT remains the most popular diagnostic scan, and MRI is the best follow-up scan.*

**Figure 3:**
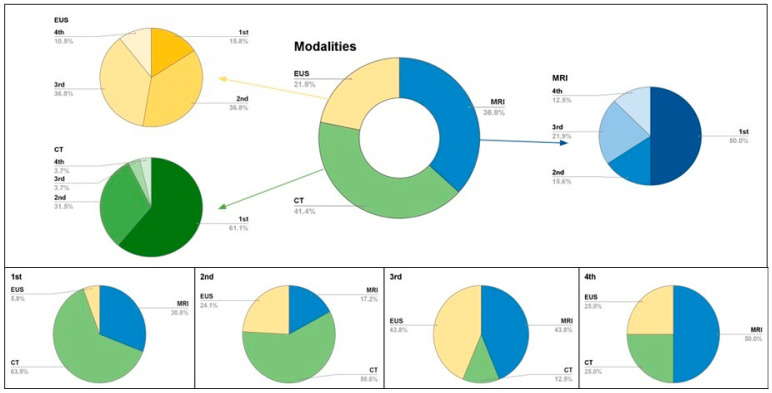
Multi-modality contributions to pNET surveillance, in consecutive order. The top box shows popularity among scans in surveillance is reflected in the doughnut chart with specific modality divisions. These are then divided into initial or follow-up orders as represented in 2D pie charts. MRI (blue), CT (green), EUS yellow. The bottom figure shows proportion of modalities contributions in pNET surveillance.

**Proof** **of** **Theorem** **1.** Figure 1, Figure 2 and Figure 3 and our inter- and intra-reader analyses show the evolving diagnostic benefits of CT and MRI over time. □

1. The sensitivity for pancreatic head tumor detection is higher than in the tail.
2. To provide optimal care for patients with rare pNETs, multidisciplinary teams of healthcare professionals need to remain updated on the latest impacts of clinical imaging trends and aware of changes in the displaying of radiological features as a result of technical advances. Given the non-specific clinical symptoms and the overlap with other gastrointestinal conditions in suspected syndromic or disparate hormonally functional pNETs, the detection of millimeter-sized tumors in this deep-seated organ requires expertise. CT is recommended as the initial imaging modality, with MRI follow-ups.
3. Our study shows CT’s dominance as the initial scan for pNET detection and an increasing preference for MRI and EUS as follow-ups. These patterns emphasize CT reliance as the primary modality for pNETs, supported by satisfactory inter-reader and intra-reader reliability for CT.
4. This study also found that three advanced radiology fellowship-trained readers performed well in the preoperative detection of pNETs, with better detection on CT compared to MRI, especially with recent technical advances.
5. However, intra-reader reliability was suboptimal, addressing the need for improvement in both CT and MRI.

## 4. Discussion 

### 4.1. Observational Trend Findings

In this single-institution retrospective study of primary modality-related tumor localization in syndromic patients, CT remained the primary modality over both decades, while MRI utilization soared exponentially during the study period (Figure 1). EUS was primarily used as the last resort if neither CT nor MRI could be indicated, but received 7% increase in popularity in the second decade of this study. On the other hand, EUS, as a more invasive direct approach, has increased in popularity for nonfunctional tumors in the most recent decade.

Syndromic Cases: MDCT was historically used as the primary modality in 88% of syndromic cases; however, it had a 38% reduction in utility for symptomatic patients. Meanwhile, MRI’s utility increased from 12% to 43%. EUS, which had no utility in the first decade for pNET detection, grew in popularity by 7% in symptomatic patients during the second decade. CT remains the imaging modality of choice, but there is a growing role of EUS in localizing pancreatic lesions in syndromic patients.

Non-Syndromic Cases: Our data showed a different perspective for non-syndromic patients.

The use of MDCT as the primary modality for pNET detection decreased by 11% from the first to the second decade (72% to 61%). MRI and EUS combined gained a remarkable increase in use, from 28% to 39%. Overall, CT usage is still prevalent, but MRI is being increasingly used. The rationale behind this shift is unclear, but it may be due to limiting exposure to radiation if patients are being imaged repeatedly for other issues.

### 4.2. Concurrent Clinical Updates in a Review of the Literature

#### 4.2.1. Evolution in Pathology Staging and Diagnosis

Our study’s context differs from earlier research, focused on inconspicuous pNET features [10,11] or mimicking features [12] pre-surgically. The seventh edition of the AJCC staging system in 2010 separated pNETs from pancreatic adenocarcinoma, but carcinomas staging criteria are still directly applied to pNETs in many studies [13,14]. However, the most recent updates in the AJCC and TNM staging system for size and symptom classification were implemented in our evaluations (Table 1) for better interpretation [15]. Studies focused on multiple endocrine neoplasia type 1 (MEN I) in patients with pNETs showed disagreements in relation to imaging priorities [16,17,18,19,20]. Other studies have considered MRI as a last resort for patients with a history of iodinated CT contrast allergies or when ultrasound was unable to identify the tumor, as shown by the following 2002 study. In addition, at a time when MRI was less available in regions like Canada and fluorodeoxyglucose positron emission tomography (FDG-PET) was not widely used, ultrasound was discussed to be the best screening alternative for pancreas screening [21].

#### 4.2.2. Surgical Management Advances

Prior to 2012, resection was recommended for all functioning pNETs and localized nonfunctional pNETs (non-syndromic in our study) for tumors larger than 2 cm [22]. This led to controversy in subsequent years, particularly regarding the management of small tumors [23,24], and which patient populations are safe for observing pNETs [25]. The North American Neuroendocrine Tumor Society (NANETS) recommends observation for PNETs < 1 cm, while suggesting individualized management based on age, comorbidity, growth, grade, extent of needed surgery, and patient preference for tumors between 1 and 2 cm [3].

#### 4.2.3. Advances in MR Imaging Techniques [26]

Advances in image reconstruction and motion artifact reduction, with the help of artificial intelligence (AI) technologies such as deep learning (DL) algorithms, have been the most significant developments in MRI over the past decade [27,28,29,30], while the advent of EUS and molecular imaging (such as 68Ga-DOTA-peptides for PET) has shown promise in sensitive detection of pNETs [31], particularly for lesions ranging from 2 to 5 mm, due to the proximity of the endoscope to the lesion. However, in a 2012 prospective study, in pancreatic tumors > 1 cm, 9/57 (15.7%) tumors were missed by EUS, and 11/57 (19.3%) tumors were missed by MRI [29]. Nevertheless, a majority of experts continue to recognize CT as a reliable modality, even in the earlier stages of detection [32,33]. MRI offers a non-ionizing alternative in multiple-imaging follow-up studies [25,34,35], while EUS performance is dependent on operator expertise. In addition to CT, MRI, and EUS, imaging based on somatostatin-labeled analogs, including 68Ga-DOTATATE positron emission tomography/CT (PET) and 111In-Octreotide (Oct), present novel approaches to pNET detection that are more resource-intensive. Their utilization has been limited in our institutional imaging workup for suspected pNETs, and therefore, these modalities were not included in our study. The routine incorporation of these techniques into clinical practice remains a topic of discussion [36,37]. Recent advancements in the understanding of pNETs have been driven by ongoing research into their molecular and genetic characteristics. Moving forward, it is crucial to understand the basics of patterns and stereotypes to establish the optimal evidence-based approach to care.

## 5. Conclusions

Our two-decade cohort from a high-volume, tertiary-care institution provided us with valuable insights for comparison and decision making in relation to the management of pNETs.

We conducted an inter-reader analysis involving three radiologists, individually evaluating the same images, and an intra-reader analysis where each radiologist reviewed random images at different time points to evaluate conventional multi-modality approaches. This evaluation provided us with an in-depth assessment of CT and MRI, which have consistently remained the most effective non-invasive imaging techniques across all clinical settings, regardless of technological advancements, preferences, and availability over time.

Computed tomography plays the leading role in the detection of pNETs, regardless of whether patients present with symptoms. Despite advancements in imaging technologies which have diminished the relative utility of CT compared to other modalities, it remains the primary initial imaging tool for detecting both symptomatic and non-syndromic tumors. Magnetic resonance imaging (MRI) has advantaged follow-up assessments, functional imaging which targets somatostatin receptors (SSTRs) may offer potential value in staging, or later phases in the management process.

## Figures and Tables

**Table 1:** Patient demographics, outcomes and imaging characteristics that influence approach to patients with pNETs.

Studied Characteristics	Syndromic	Non-Syndromic	Overall
Number of patients	*N* = 35	*N* = 40	*N* = 75
Presented with metastases	*N* = 9	*N* = 7	*N* = 16
Mean age ± SD	53.43 ± 16.36	55.97 ± 18.10	54.7 ± 17.23
Gender	5 Male, 30 Female	22 Male, 18 Female	27 Male, 48 Female
<2 cm	*N* = 23	*N* = 17	*N* = 40
2–4 cm	*N* = 6	*N* = 9	*N* = 15
>4 cm	*N* = 6	*N* = 13	*N* = 19
Tumor site	Head: *N* = 15 Neck: *N* = 3 Body: *N* = 5 Tail: *N* = 12	Head: *N* = 8 Neck: *N* = 3 Body: *N* = 7 Tail: *N* = 13	Head: 32% Neck: 9% Body: 18% Tail: 41%
MRI	*N* = 16 13 detections in initial scan	*N* = 16 8 detections in initial scan	*N* = 32
CT	*N* = 21 19 detections in initial scan	*N* = 42 37 detections in initial scan	*N* = 63
EUS	*N* = 17 11 detections in initial scan	*N* = 18 5 detections in initial scan	*N* = 35
Surgery	Ex-lap: *N* = 29 Whipple: *N* = 24	Ex-lap: *N* = 20 Whipple: *N* = 2	Ex-lap: *N* = 49 Whipple: *N* = 26

## Data Availability

Data can be provided for reviewers in cases of necessity, according to our institutional agreement policies.
